# Retraction: Glyphosate Use Predicts ADHD Hospital Discharges in the Healthcare Cost and Utilization Project Net (HCUPnet): A Two-Way Fixed-Effects Analysis

**DOI:** 10.1371/journal.pone.0137489

**Published:** 2015-08-21

**Authors:** The PLOS ONE Staff

The publisher wishes to retract this paper because it was published in error. This paper was editorially rejected following peer review and consultation with the Editorial Board. Unfortunately, the production process was erroneously completed on this paper resulting in publication, and the publisher apologizes for this mistake.

## References

[1] Fluegge KR, Fluegge KR (2015) Glyphosate Use Predicts ADHD Hospital Discharges in the Healthcare Cost and Utilization Project Net (HCUPnet): A Two-Way Fixed-Effects Analysis. PLoS ONE 10(8): e0133525 doi:10.1371/journal.pone.0133525 26287729 10.1371/journal.pone.0133525 PMC4543553

